# The impact of virtual learning on students’ educational behavior and pervasiveness of depression among university students due to the COVID-19 pandemic

**DOI:** 10.1186/s12992-022-00863-z

**Published:** 2022-07-14

**Authors:** Fatima M. Azmi, Habib Nawaz Khan, Aqil M. Azmi

**Affiliations:** 1 Department of Mathematics and Sciences, College of Humanities and Sciences, Prince Sultan University, Riyadh, Saudi Arabia; 2 Department of Statistics, University of Science and Technology, Bannu, KPK Pakistan; 3 Department of Computer Science, College of Computer and Information Sciences, King Saud University, Riyadh, Saudi Arabia

**Keywords:** COVID-19, Depression, Stress, Saudi Arabia, Higher education, e-learning, Quality education, SDG 4

## Abstract

**Background:**

One of the worst pandemics of recent memory, COVID-19, severely impacted the public. In particular, students were physically and mentally affected by the lockdown and the shift from physical person-to-person classrooms to virtual learning (online classes). This increased the prevalence of psychological stress, anxiety, and depression among university students. In this study, we investigated the depression levels in Saudi Arabian university students who were learning virtually because of the COVID-19 pandemic and examined its impact on their educational proficiency.

**Methods:**

The study focused on two points: first, examining the depression levels among undergraduate students in Saudi Arabia, by adapting the Zung (Self-Rating Depression Scale) questionnaire. Second, whether there is an association between the levels of depression and various distress factors associated with virtual (online) learning resulting from the COVID-19 pandemic and its impact on students’ educational behaviors. The questionnaire was prepared using a monkey survey and shared online, via email, and on WhatsApp groups, with participants in two universities, a public and private university in the largest city of Saudi Arabia. A total of 157 complete responses were received. Data were analyzed using SPSS-24, the chi-square test, descriptive statistics, and multilinear regression.

**Results:**

The results indicated that three-fourths of the university students suffered from different depressive symptoms, half of which had moderate to extreme levels of depression. Our study confirmed that a boring virtual (online) learning method, stress, fear of examinations, and decreased productivity were significantly associated with increased depression. In addition, 75% and 79% of the students suffered from stress and fear of examinations, respectively. About half of the students were associated with increased depression. The outcome also indicated that female students experienced extreme depression, stress, and fear of examinations more than males.

**Conclusion:**

These findings can inform government agencies and representatives of the importance of making swift, effective decisions to address students’ depression levels. It is essential to provide training for students to change their educational experience mindset, which might help decrease "depression and stress-related growth." There is also a need to search for a better virtual teaching delivery method to lessen students' stress and fear of examinations.

## Background

On March 11, 2020, the World Health Organization (WHO) declared the highly contagious coronavirus (COVID-19) a global pandemic [1]. As the cases of COVID-19 increased, China, and many other countries practiced partial or complete lockdowns. It is estimated that this drastic measure helped save 3 million lives across 11 European nations [2]. Toward the end of January 2022, the total number of confirmed COVID-19 cases worldwide was 360,578,392, and 5,620,865 confirmed deaths. The number of people who received vaccination doses globally was 9,679,721,754 [3].

To contain the virus, the lockdown caused academic disruptions. This resulted in the indeterminate closure of schools, universities, various institutes, shopping malls, and centers of economic activities [4, 5]. Repetitive activities, transfer of educational mode to distance (virtual) learning, and change in social life amplified the prevalence of psychological stress, anxiety, depression, and acute stress reactions among university students [6]. Sociodemographic factors associated with low mental health include financial constraints, old age, infection risk, and fear of losing a relative or friend. In addition, COVID-19 pandemic-related educational stress may be attributed to (in no particular order): (a) transformed teaching and assessment methods; (b) skepticism about university education; (c) technological worries about online courses [7, 8]; (d) uncertainty about the future because of academic disruptions; (e) fear of failing examinations; (f) inability to concentrate during lectures, and many more factors. All these factors have been detected in universities across the world [9, 10]. A global study that inspected students’ experiences in about 62 different countries, including a university in the United States, found that students expressed worries about their academic achievements and professional careers and feelings of dullness, anxiety, and frustration [9]. Students in China also reported increased sadness, anger, anxiety, and fear [11]. The occurrence of depression, psychological distress, and anxiety from pandemics differed from country to country. A study in Italy reported that 15.4% of Italians suffered from extremely high levels of depression, 12.6% were highly stressed, and 11.5% were highly anxious [12]. In Malaysia, it was reported that severe to extremely severe levels of depression and anxiety were found in 9.2% and 13.2% of the subjects, respectively. Moderate stress was found in 9.5% of subjects, and severe to highly severe stress was found in 6.6% of subjects [13]. Furthermore, students in Switzerland manifested a decrease in social interface and higher levels of stress, anxiety, and loneliness [14]. Adults have also reported declining physical activity, while food eating increased during pandemic quarantine periods compared to previous times [15].

The first COVID-19 case appeared in the Kingdom of Saudi Arabia (KSA) on March 2, 2020 [16], while the lockdown was imposed on March 8, 2020. To keep students on track due to the pandemic, the education delivery mode was switched to virtual learning. It has been over one year since teaching was transferred online, and many countries worldwide have tried to revert to the standard path of education by opening schools and universities. Although the COVID-19 vaccine is available worldwide, some countries are still practicing lockdown because of the appearance of several more contiguous variants of the coronavirus, such as Delta, a SARS-CoV-2 strain that was first spotted in India [17]. The spread of COVID-19 presents a serious risk; in mid-April 2022, the confirmed cases in KSA were 751,717, out of which 736,910 had recovered, and 9,055 deaths were recorded [18].

The psychological consequences of COVID-19 have been observed and described in KSA. Al-Hanawi et al. [19] reported different levels of distress in 40% of the general Saudi population because of COVID-19. Moreover, Alkhamees et al. [20] reported moderate to severe psychological effects in 23.6% of the general Saudi population. In another study of the influence of the COVID-19 pandemic on Saudi Arabian residents, Alyami et al. [21] stated that the percentages of mild, moderate, moderately severe, and severe levels of depression were 41%, 20%, 6.2%, and 3.2%, respectively. Furthermore, Khoshaim et al. [22] reported that about 35% of students experienced moderate to extreme anxiety levels. Azmi et al. [23] observed that 75% of students suffered from various levels of depression, while 41% suffered from low levels of self-esteem.

Likewise, another study found that 35% of students in the western and northern regions of KSA had high rates of distress [24]. Following the observed rise of psychological disorders, the authorities posted health messages and distributed procedures to the public. For example, during the pandemic, the Saudi Center for Disease Control and Prevention (CDC) [25] supplied a precautionary manual for mental and social health focused on prevention, pressure, and fear control. From the foregoing, the COVID-19 pandemic has had a severe impact on the physical and mental health of the public in general and students in particular, as university students are among those most severely affected by the COVID-19 pandemic.

In this study, we investigated the depression levels of university students in Riyadh, the capital and largest city in KSA, who were learning virtually because of the COVID-19 pandemic. We also assessed the impact of virtual learning on their educational behaviors. The following questions were explored during the investigation:

1. What are the levels of depression among university students?
2. What is the impact of virtual learning on students’ educational behaviors and what are the relationship between depressive symptoms they exhibited and virtual learning?

To answer the second question, we explored the relationship between the levels of depression and various distress factors associated with virtual learning because of the COVID-19 pandemic and its impact on students’ educational behaviors. These factors were divided into two main categories: Category 1 dealt with factors relating to how virtual learning has affected students’ feelings from an educational perspective. Category 2: dealt with factors relating to how virtual learning affected students’ understanding of subjects/learning materials.

Once we ascertain the current levels of depression and their impact on students’ educational behavior, we may embark on helping them cope with the extraordinary situation. Hopefully, this will help lower their elevated depression levels. Furthermore, we hope our study will guide policymakers in searching for innovative ways of online teaching to make learning less stressful and more productive.

## Methods

### Design and sampling procedure

This study examines depression levels and investigates virtual learning-related distress factors, which might predict the increased level of depressive symptoms among university students in Riyadh City during the COVID-19 pandemic.

### Research design

We conducted a descriptive survey-based study to obtain responses from students at large universities in Riyadh, the capital of KSA. The total size of the target population of the city of Riyadh is about 7 million [26]. The sampled population of both universities’ undergraduate students was approximately 0.027 million (27,000). The male-to-female ratio of undergraduate students at King Saud University (KSU) is about 67%: 33%; the male-to-female ratio of undergraduate students at Prince Sultan University (PSU) is about 28%: 72%, as this is a female-dominated university. Since the sampled population was largely heterogeneous, we minimized the heterogeneity by dividing the given population into sub-populations to obtain sampling units that are homogeneous internally and heterogeneous externally. Hence, we used a stratified random sampling technique, which is more appropriate than other sampling techniques for obtaining better estimates of the parameters of interest. To ensure the efficiency of the estimates, we used the proportional allocation technique to determine the sample size.

A Monkey survey was used to prepare the questionnaire, following the approval of PSU’s Institutional Review Board. The questionnaire included demographic questions, such as gender, age, and college. Zung’s Self-Rating Depression Scale (ZSDS), with 20 items on a 4-point Likert scale, was used to measure depression. The questionnaire also had questions to address distress factors associated with virtual learning because of the COVID-19 pandemic. The students were asked to read all the questions carefully and answer them.

The survey was written in English and Arabic side by side. A subject expert translated the questionnaire from Arabic to English. Thereafter, five more experts checked the same questions for more corrections and authenticity. The actual online survey took place from March to April 2021. The survey was voluntary, and the informed consent of the students was sought. We received reasonable responses from the students; however, we also received some incomplete responses. The missing/incomplete responses were discarded from the study so that the estimated results were not compromised. The valid responses received from males and females were 49.7% and 50.3%, respectively.

### Measuring instruments

Demographic data and personal characteristics, such as age, gender, and area of study, were recorded.

### Depression measure

The ZSDS was used to measure the levels of depression. The tool is a 20-item self-reporting assessment device used for measuring depression levels [27, 28]. This is divided into 10 positively worded and 10 negatively worded items. The latter items were reversely scored. Each item was scored on a Likert-type scale as follows: 1 = *Never*, 2 = *Sometimes*, 3 = *Often/most of the time*, and 4 = *Always*. The total raw scores ranged from 20–80, and when converted into the depression index (termed "ZSDS index"), the range becomes 25–100. To determine the level of depression, we classified the ZSDS index into four classes (levels). Therefore, ZSDS index scores were considered "normal" from 25–49, "Mildly Depressed,” from 50–59, “Moderately Depressed” from 60–69, and “Severely Depressed” from 70 and above [27]. In [29], the author translated the ZSDS measure into Arabic and further validated it. Question 6, “I still enjoy sex,” was deemed offensive religiously and culturally. Therefore, it was rephrased to “I enjoy looking at, talking to, and being with attractive women/men,” which is culturally more appropriate. The accuracy of the new version was verified in [29]. The Arabic and English languages were used side by side to prepare the questionnaire. The Cronbach’s alpha coefficient of this study was 0.87, showing high internal consistency.

### Data on distress factors associated with virtual learning

Data on distress factors associated with virtual learning due to the COVID-19 pandemic were divided into two categories. The first category dealt with questions on how virtual learning due to the pandemic affected students’ feelings from an educational perspective and caused a) lack of motivation/boredom, b) stress, c) worry and fear of exams, and d) decreased productivity. The second category dealt with questions on virtual learning and its effect on students’ understanding of subjects/materials, such as a) It needs more self-effort to understand, b) It made learning and understanding harder for them, c) They need more time to understand the subject, i.e., the understanding pace became slower, d) Virtual learning is boring, and e) they had difficulty solving problems in academic subjects and writing down the solutions correctly. The answer to each question was either “Yes” or “No.”

Finally, the questionnaire had an open-ended question that offered students a chance to express in their own words how the lockdown and virtual teaching had affected their educational advancement.

### Data analysis

Data were analyzed using IBM SPSS version 24 software. The categorical variable demographic data were analyzed descriptively to determine the essential characteristics of the sample and were presented as counts and percentages. The level of depression index among university students in Riyadh, and its association with gender, age, and their field of education, was analyzed using the chi-square test and descriptive statistics. Multilinear regression analysis was performed to investigate the connection between depression levels and various factors associated with virtual learning due to the COVID-19 pandemic. The statistically significant level was set at $p \le 0.05.$

## Results

### Demographic characteristics

The total number of participants was 157 university students. Table 1 shows the demographic characteristics of the participants.

**Table 1:** Demographic characteristics of the respondents

Variable	Frequency	Percentage
**Gender**		
Male	78	49.7%
Female	79	50.3%
**Age group**		
< 25 Years	151	96%
$\ge 25$ Years	6	4%
**College**		
Computing and IS	103	65.5%
Business Administration	37	23.5%
Engineering and Science	10	6%
Others	7	5%

### Students’ levels of depression and demographic variables

In the univariate analysis, chi-square tests were used to determine the associations between students’ demographic variables and the ZSDS level. Table 2 displays the association between depression levels with gender, age, and college. Among the demographic variables, only the association with gender was statistically significant at ${\chi }^{2}$ = 20.229, and *p* < 0.001, while the association with age and college was not significant. A total of 74.4% of the students had various levels of depression. Of these, 37%, 21.7%, and 16% had mild, moderate, and severe depression levels, respectively. In addition, females (28%) had more depressive symptoms than males (4%).

**Table 2:** Association between depression levels and students’ demographic variables in Saudi Arabia

**Variable**	**ZSDS**					
	**Normal (%)**	**Mild (%)**	**Moderate (%)**	**Severe (%)**	**Chi-square**	**P-Value**
**Gender**					20.229^a^	< 0.001
Male	26 (33.3%)	34 (43.6%)	15 (19.2%)	3 (3.8%)		
Female	14 (17.7%)	24 (30.4%)	19 (24.1%)	22 (27.8%)		
**Total**	40 (25.4%)	58 (37%)	34 (21.7%)	25 (16%)		
**Age Group**					2.241	0.524
< 25 years	37 (24.5%)	57 (37.5%)	33 (22%)	24 (16%)		
$\ge 25$ years	3 (50%)	1 (16.7%)	1 (16.7%)	1 (16.7%)		
**College**					3.181	0.786
Business	9 (24.3%)	13 (35.1%)	9 (26.5%)	6 (24%)		
Computing and IS	25 (24.3%)	40 (38.8%)	20 (19.4%)	18 (17.5%)		
Others	6 (35.2%)	5 (29.4%)	5 (29.4%)	1 (6%)		

^a^0 cells (0.0%) have less than 5 expected counts

### Educational distress factors associated with virtual learning and descriptive statistics

The factors related to virtual learning sequel to the COVID-19 pandemic, and its impact on students’ educational behaviors were divided into two categories. Questions on virtual learning's effect on students' feelings from an educational perspective (Category 1) had four items, each with a "Yes" or "No" answer. Likewise, questions on virtual learning and its effect on students’ understanding of the subjects/materials (Category 2) had five items, each with a “Yes” or “No” answer. Table 3 demonstrates the descriptive statistics. In the first category, the highest percentage was feeling worried and having a fear of exams (79%), followed by stress (75.2%), lack of motivation, and decreased productivity (70%, each). In the second category, the highest percentage was 78%, who felt they had to put extra self-effort into understanding and studying.

**Table 3:** Educational distress factors associated with virtual learning due to the COVID-19 pandemic and descriptive statistics

Description		YES N (%)	NO N (%)
**C1. Virtual learning and its effect on students’ feelings from an educational perspective**			
Lack of Motivation	Feeling unmotivated/ bored/Lazy/unproductive	110 (70%)	47 (30%)
Stress	Feeling overwhelmed and stressed	118 (75.2%)	39 (24.8%)
Worry and exam fear	Feeling worried and fearful of exam/Panicked	124 (79%)	33 (21%)
Decreased Productivity	Feeling decreased learning and being unproductive	110 (70%)	47 (30%)
**C2. Virtual learning and its effect on students’ understanding of the subjects/materials**			
Extra self-effort	The need to put extra effort to understand the lecture	122 (77.7%)	35 (22.3%)
Need to study harder	The study is more challenging when taught virtually	117 (74.5%)	40 (25.5%)
Learning is slower than standard	The need to spend more time to understand the material/topic	115 (73.2%)	42 (26.8%)
Learning is boring	Virtual learning is not exciting or fun	100 (63.7%)	57 (36.3%)
Difficulty in solving problems	Find difficulty in solving problems and submitting properly written answers	92 (58.6%)	65 (41.4%)

Furthermore, 74.5% felt that virtual learning was more challenging for them to understand than physical learning. In addition, 73% said virtual learning was slow and extra time was needed to understand and learn the concepts, while 64% found it boring. Finally, 58.6% had difficulty solving problems and submitting properly written answers (for math and computer science subjects).

### Distress factors related to virtual learning and depressive symptoms

Multilinear regression analysis was used to study whether various distress factors related to virtual learning can influence depressive symptoms among students.

The first category, which dealt with students’ feelings from the educational point of view, hypothesized that lack of motivation, stress, worry/fear of examinations, and decreased productivity would significantly impact the development of depressive symptoms among students.

Multi-regression analysis was used to test the hypotheses, with the Zung depression index as a dependent variable. The results show that 24.6% of the variance in Zung’s depression index can be accounted for by four predictors, collectively$, F(4, 152) = 12.414, p < 0.001$. Looking at the unique individual contribution of the predictors, the result shows that worry and fear of exams ($\beta =0.290, t=3.589, p<0.001)$, stress ($\beta =0.202, t=2.566, p=0.011<0.05)$, and decreased learning amount and not being productive ($\beta =0.211, t=2.783, p=0.006<0.05)$, statistically significantly contributed to worsening depressive symptoms. The predictor, feeling lack of motivation, did not significantly impact developing depressive symptoms.

The second category dealt with virtual learning and its effect on students’ understanding of the subjects/materials. It was hypothesized that the need for extra self-effort to understand the subject, learning became harder, learning became slower, learning was boring, and difficulty in solving problems and writing answers properly would have a statistically significant impact on developing depressive symptoms among students.

Multi-regression analysis was used to test the hypotheses, with Zung’s depression index as a dependent variable. The test showed that 13% of the variance in Zung's depression index can be accounted for by the five predictors, collectively$, F(5, 151) = 4.505, p < 0.001$. Looking at the unique individual contribution of the predictors, the result shows that learning is not much fun or exciting ($\beta =0.250, t=3.060, p=0.003<0.05)$, and facing difficulty in solving questions and writing answers properly ($\beta =0.176, t=2.067, p=0.05<0.05)$, were statistically significantly associated with worsening depressive symptoms. While the other three predictors, learning became harder, learning became slower, and the need to put extra self-effort did not contribute significantly to depressive symptoms, as shown in Table 4.

**Table 4:** Results of multi-regression analysis

Factors	B	SE	$\beta$	t	*P*-value
**Virtual learning and its effect on students' feelings from an educational point of view**					
Lack of Motivation	$-0.493$	1.916	$-0.19$	$-0.257$	0.797
Stress	2.724	1.061	0.202	2.566	**0.011**
Worry and exam fear	2.074	0.578	0.290	3.589	** < 0.001**
Decreased Productivity	0.767	0.275	0.211	2.783	**0.006**
**Virtual learning and its effect on students’ understanding of the subjects/materials**					
Extra self-effort	$-0.256$	$2.178$	$-0.009$	$-0.118$	$0.906$
Need to study harder	0.775	1.200	0.058	0.646	0.519
Learning is slower compared to physical teaching	$-0.112$	0.817	$-0.013$	$-0.137$	0.891
Learning is boring	1.519	0.497	0.250	3.060	**0.003**
Difficulty in solving homework questions	0.832	0.403	0.176	2.067	**0.040**

*B* regression coefficient, *SE* Standard Error, the dependent variable is Zung’s depression index

Furthermore, we explored two distress factors, stress, and worry/fear of exams, which contributed statistically significantly to worsening depressive symptoms. Using the chi-square test, we examined the association of the distress factors with depression levels; that is, what extent does stress or worry/fear of exams contribute to moderate or severe depression. The results showed a statistically significant association between stress and moderate to severe levels of depression ( ${\chi }^{2}$ = 17.179, and *p* < 0.001). Likewise, there was a statistically significant association between worry/fear of exams and moderate to severe levels of depression ( ${\chi }^{2}$ = 30.236, and *p* < 0.001), Table 5.

**Table 5:** Chi-square test for association between a few distress factors and depression levels

**Factors**	**ZSDS Levels**			**Chi-square**	***P*-Value**
	**Normal (%)**	**Mild (%)**	**Moderate to Severe (%)**		
**Stress**	24 (20%)	39 (33%)	55 (47%)	17.179^a^	< 0.001
**Worry and fear of exams**	21 (17%)	45 (36%)	58 (47%)	30.236^a^	< 0.001

^a^0 cells (0.0%) have less than 5 expected counts

The association between stress or worry/fear of exams and gender was examined using the chi-square test. There was a statistically significant association between these two factors and gender, with more females having higher stress levels (54%) than males (41%). Also, worry/fear of exams manifested in 60% of females and 40% of males during virtual learning, sequel to the COVID-19 pandemic. The results are presented in Table 6.

**Table 6:** Chi-square test for association between a few distress factors and gender

Factors	Male (%)	Female (%)	Total	Chi-square	*P*-Value
**Stress**	48 (40.7%)	70 (59.3%)	118 (75%)	15.404^a^	< 0.001
**Worry and fear of exam**	50 (40%)	74 (60%)	124 (79%)	20.670^a^	< 0.001

^a^0 cells (0.0%) have less than 5 expected counts

### Open-ended questions

The questionnaire ended with an open-ended question, in which students were asked to write in their own words how the lockdown has affected their educational advancement. Excerpts of the negative comments from students are outlined below:

“Virtual teaching and exam resulted in increased cheating."

“Virtual teaching caused difficulty in understanding the subject, which resulted in lowering my grades.”

"I have to sit in the same room with my siblings while learning online, as my home is small. So, I cannot concentrate at all; it just makes me very frustrated.”

From their comments, it is clear that a virtual learning environment is entirely different from a physical classroom teaching environment where exams are conducted with invigilators proctoring.

Significantly few students provided positive comments.

"Virtual teaching made me understand better and increased productivity and my grades."

## Discussion

In this study, we investigated the severity of depressive symptoms among university students while learning virtually because of the COVID-19 pandemic and its impact on educational behaviors in KSA We collected samples from different universities in Riyadh. The total number of complete responses was 157. The Zung Self-Rating Depression measure was used to measure depression levels. Our results indicate that 75% of the students suffer from different levels of depression (37%, 22%, and 16% of the students reported mild, moderate, and extremely severe levels of depression, respectively). This result is consistent with an American study, which reported that 44% of students in the USA experienced an augmented level of depressive thoughts [30].

The association between the levels of depression and various distress factors associated with virtual learning due to the pandemic and its impact on students’ educational behaviors was explored using multilinear regression. These factors are divided into two main categories: Category 1: These factors relate to how virtual learning has affected students’ feelings from an educational perspective. This consists of four items: lack of motivation, stress, worry/fear of exam, and decreased productivity. Category 2 factors relate to how virtual learning has affected students’ understanding of the subjects/materials. This category has five items, including need of extra self-effort, need to study harder, learning is slower, virtually learning is boring, difficulty in solving problems, and writing properly.

Consistent with our hypotheses, we confirmed that stress, worry/fear of examinations, and decreased productivity were significantly associated with an increased level of depression. Another recognized factor that contributes significantly to a higher risk of developing depressive symptoms among university students is that virtual teaching and learning becomes boring. Furthermore, students faced difficulty in solving mathematics and science problems and writing the answers properly due to online teaching. A few other factors, such as lack of motivation, learning became more complex and slower, and the need to put extra self-effort contributed to developing depressive symptoms.

Our results indicate that 75% of the students suffer from stress, and about half (47%) have high levels of depression. This is consistent with the results in [13]. Our findings also indicate that 79% of the students suffer from fear of exams, and about half of them (47%) experience moderate to severe levels of depression. It is usual for some students to have worries and fear for exams; however, it is highly unusual for more than three-fourths of the students to experience fear and worry. This is a clear indication that the changed mode of lecture delivery and exam administration because of COVID-19 has a significant role in raising depression levels among university students. Our findings indicate that a higher percentage of females experience extreme levels of depression than males (28% of females compared to only 4% of males), stress (59% females, vs. 41% males), and worry/fear of exams (60% females, vs. 40% males). This finding is consistent with many studies concerning college students, in which females were at a higher risk of suffering psychologically during virtual learning because of the COVID-19 pandemic [9, 31–33]. Another study showed that Vietnamese female students had a higher percentage of depression compared to male students [34]. Furthermore, Huange et al. [35] reasoned that Chinese females experienced more anxiety than males during the COVID-19 pandemic. Thus, we assert that feamles are more commonly inclined toward depression and anxiety disorders than males [36].

The results of the open-ended responses demonstrated the students’ frustration and stress relating to online learning. In contrast, very few students positively indicated that online learning and studying from home felt relaxing.

## Conclusion

COVID-19 has been a catastrophic experience. Although it has largely subsided, new variants are causing apprehension among health officials. Our research found that 75% of university students in Saudi Arabia suffer from some degree of depression. Half of these students showed moderate to extreme levels of depression. This is greater than the expected depression level in the overall population. Our study confirms that stress, worry, and fear of examinations, decreased productivity, and the fact that virtual learning is boring are significantly associated with increased depression. Our findings also indicate that 75% (79%) of the students suffer from stress (fear of exams), and that about half of them have increased levels of depression. It should be noted that the students are 18–24 year olds. This is consistent with the study [22], which found that psychological distress, stress, and anxiety were higher in the younger age group during the COVID-19 pandemic.

Remarkably, more female students experienced extreme depression, stress, and fear of exams than male students. This result supports previous reports that females were at higher risk of psychological distress during the COVID-19 pandemic [9, 31–33].

Our observation calls for instant attention and sustenance for students. There is a requirement to explore potential coping policies that have been shown to be effective during pandemics [37]. The results of our research might direct policymakers to develop distress management protocols as part of their policy for dealing with future pandemics [38]. It is essential to provide training for students to redirect their educational experience mindset to focus on the “bright side” and expand instances that may guide "depression and stress-related growth.” A flexible mindset can also help students adapt to new ways of learning and developing tremendous gratitude for life. In addition, there is a need to explore better online teaching delivery methods to lower students’ stress and fear of exams.

### Study strengths and limitations

The strength of this study is that it was conducted after students had received virtual teaching for more than one year because of the Pandemic. Therefore, this study accurately reflects students’ depression levels and how these impact their educational behaviors in KSA.

Furthermore, the study was conducted in Riyadh, the capital of KSA, hence our study sample is more reflective of the Saudi student population. Moreover, the depression assessment tool for the study, the Zung Self-Rating Depression Scale, is a reliable, universally acceptable scale.

The limitation of our study is that the sample was not randomly selected from all university students, as a convenience sampling method was used.

## Data Availability

The raw data supporting the results of this study will be made available by the corresponding author without undue reservation.
